# P-Glycoprotein Activity at Diagnosis Does Not Predict Therapy Outcome and Survival in Canine B-Cell Lymphoma

**DOI:** 10.3390/cancers14163919

**Published:** 2022-08-13

**Authors:** Valéria Dékay, Edina Karai, András Füredi, Kornélia Szebényi, Gergely Szakács, Péter Vajdovich

**Affiliations:** 1 Department of Clinical Pathology and Oncology, University of Veterinary Medicine Budapest, István Utca 2, H-1078 Budapest, Hungary; 2 Institute of Enzymology, Research Center of Natural Sciences, Eötvös Loránd Research Network, Magyar Tudósok Körútja 2, H-1117 Budapest, Hungary or; 3 Institute of Cancer Research, Medical University of Vienna, Borschkegasse 8A, A-1090 Vienna, Austria

**Keywords:** canine, lymphoma, B-cell type, therapy resistance, P-glycoprotein

## Abstract

**Simple Summary:**

Clinical experience in human and canine clinics shows that following initial response to treatment, drug-resistant cancer cells frequently evolve and eventually, most tumors become resistant to all available therapies. The most straightforward cause of therapy resistance is linked to cellular alterations that prevent drugs from acting on their target. Drug efflux mediated by the ABC transporter P-glycoprotein (P-gp) contributes to unfavorable treatment outcome in several human malignancies. Here, we characterize a large cohort of canine B-cell lymphoma patients followed for over 7 years. We show that the intrinsic P-gp activity of tumor cells characterized at the time of diagnosis is not predictive for therapy outcome. Our results highlight the complexity of clinical drug resistance mechanisms and suggests that the relevance of P-gp in acquired resistance should be further investigated by the continuous monitoring of tumor cells during treatment.

**Abstract:**

Various mechanisms are known to be involved in the development of multidrug resistance during cancer treatment. P-glycoprotein (P-gp) decreases the intracellular concentrations of cytotoxic drugs by an energy-dependent efflux mechanism. The aim of this study was to investigate the predictive value of P-gp function based on the evaluation of P-gp activity in tumor cells obtained from canine B-cell lymphoma patients at diagnosis. P-gp function of 79 immunophenotyped canine lymphoma samples was determined by flow cytometry using the Calcein assay. Dogs were treated with either the CHOP or the L-CHOP protocol, a subset of relapsed patients received L-asparaginase and lomustine rescue treatments. Among the 79 dogs, the median overall survival time was 417 days, and the median relapse-free period was 301 days. 47 percent of the samples showed high P-gp activity, which was significantly higher in Stage IV cancer patients compared to Stage II + III and V. Whereas staging was associated with major differences in survival times, we found that the intrinsic P-gp activity of tumor cells measured at diagnosis is not predictive for therapy outcome. Further studies are needed to identify the intrinsic and acquired resistant mechanisms that shape therapy response and survival in B-cell canine lymphoma patients.

## 1. Introduction

In humans, non-Hodgkin lymphoma (NHL) was responsible for 544,000 new cases and 260,000 deaths worldwide in 2020 [1], and 8540 new cases with 920 deaths in the USA in 2021 [2]. It is estimated that over 250,000 cases of canine lymphoma were diagnosed in domestic dogs in the same period. Other studies have revealed that malignant lymphoma is the second most common neoplasm in canine patients with an estimated incidence rate of 0–100/100,000 dogs, affecting all breeds and ages [3,4]. In addition to immunotherapy using the monoclonal antibody rituximab, chemotherapy is still widely used in the treatment of human lymphoma. Recognizing similarities between human and canine lymphoma has been crucial in the successful diagnosis and treatment of the disease in both species [5,6,7,8,9]. One of the most important factors limiting the efficacy of chemotherapy is drug resistance [10,11]. Drug resistant cancer cells survive treatment despite the use of the maximum tolerated doses of antitumor agents [12,13]. Multidrug resistant (MDR) cancer cells are simultaneously resistant to a variety of cytotoxic drugs with different mechanisms of action. Elevated levels of ATP-binding cassette (ABC) transporters have been reported to be linked to MDR [14]. However, the contribution of active drug efflux from cancer cells to the therapy resistance of patients has been difficult to ascertain [15]. P-glycoprotein (P-gp), a transmembrane glycoprotein that belongs to the ABC transporter superfamily, plays an important role in drug resistance by recognizing and extruding various chemotherapeutical molecules from cells [10,16]. P-gp is an independent prognostic factor in several human malignancies [17,18], and several studies suggest that it influences the treatment outcome of canine cancer including mast cell tumors [19], mammary tumors [20], pulmonary carcinoma [21], and lymphoma [22,23,24,25]. To estimate the P-gp levels, the expression can be quantified by immunohistochemistry [10,23,25], Western blotting [26], quantitative reverse transcription PCR (RT-qPCR) [27], or flow cytometry [28]. However, it can be argued that P-gp protein levels do not necessarily reflect the function of P-gp [29]. Indeed, clinical outcomes in human studies show the strongest correlation with functional measurements as opposed to the detection of MDR mechanisms at the protein or mRNA levels [14].

Fluorescence-based assays have been widely applied to measure P-gp activity. MDR cells expressing P-gp extrude nonfluorescent Calcein AM, reducing the accumulation of fluorescent calcein in the cytosol. Similarities between humans and dogs allow for the use of the same diagnostic methods, although the clinical relevance of these tests might be different in human and canine clinics. In humans, the Calcein assay has been used to assess the contribution of P-gp function to clinical MDR [30,31,32,33]. Several groups have attempted to establish the role of P-gp in the treatment outcome of canine lymphoma, with controversial results [26,34,35]. The Calcein AM assay has been tested in canine cell lines [36,37,38,39], but to our knowledge, it has not been evaluated in the clinical setting of canine lymphoma. Here, we present the correlations between the results of the Calcein assay and survival times established in a large cohort of canine B-cell lymphoma patients. 

## 2. Materials and Methods

### 2.1. Patients and Samples

A total of 79 dogs were enrolled in the study conducted between 2013 and 2020 in the Veterinary Hematology and Oncological Center, Budapest. The staging method was based on physical examination, two-sided chest X-ray, and abdominal ultrasonography in addition to blood tests including complete blood count, clinical chemistry profile, and acid–base analysis. Stages (and substages) were defined according to the scheme established by the World Health Organization (WHO) [8]. The diagnosis and immunophenotype of the tumors were determined by flow cytometry or immunohistopathology [6]. Inclusion criteria included proven cytological or histological diagnosis of high-grade lymphoma of multicentric forms, the lack of previous treatment with cytotoxic agents, complete pre-treatment clinical staging (complete blood count, clinical chemistry panel, acid–base and electrolyte assessing, urine analysis, ultrasonography, chest X-ray and bone marrow evaluation), and owner compliance with respect to cytostatic treatment and control visits. Exclusion criteria included the discontinuation of treatment, the death of the animal before starting chemotherapy and chronic diseases not related to lymphoma. The CHOP protocol consisted of doxorubicin (Adriamycin RDF/PFS injection, Pharmacia & Upjohn S.p.A. Co., Milan, Italy) 30 mg/m^2^: weeks 1, 10, 19; vincristine (Vincristine liquid injection, Gedeon Richter Co., Budapest, Hungary) 0.75 mg/m^2^: weeks 2–9 and 11–18; cyclophosphamide (Endoxan injection, Baxter Co., Pueblo County, CO, USA) 250 mg/m^2^: weeks 4, 7, 13, 16; prednisolone (Prednisolone tablet, Gedeon Richter Co., Budapest, Hungary): 2 mg/kgbw week 1, perorally (po) daily, once a day (SID), 1.5 mg/kgbw week 2 po daily, SID, 1 mg/kgbw week 3 po daily, SID, 0.5 mg/kgbw week 4, po daily, SID. The protocol was applied in 9-week cycles, repeated twice, starting and ending with doxorubicin (Table 1).

The L-CHOP or Madison–Wisconsin protocol was initiated with an L-asparaginase injection (L-Asparaginase injection, Health Biotech Ltd., Chandigarh, India) at 400 IU/kgbw IM. The remaining drugs were administered during a 25-week period as follows: vincristine 0.75 mg/m^2^: weeks 1, 3, 6, 8, 11, 15, 19, 23; cyclophosphamide 250 mg/m^2^: weeks 2, 7, 13, 21; doxorubicin: 30 mg/m^2^: weeks 4, 9, 17, 25; prednisolone: 2 mg/kgbw week 1, po daily, SID, 1.5 mg/kgbw week 2 po daily, SID, 1 mg/kgbw week 3 po daily, SID, 0.5 mg/kgbw week 4, po daily, SID (Table 2) (52).

The rescue treatment option was combination therapy with continuous L-asparaginase and lomustine (Cecenu capsules, Medac GmbH, Wedel, Germany). Asparaginase was intramuscularly injected at 400 IU/kgbw and lomustine was given at 60–80 mg/m^2^ po on the same day. This treatment was repeated every three weeks until recurrence.

All treatment protocols were administered by the team of the Veterinary Hematology and Oncological Center. Sample analysis, data collection, and curation were performed in collaboration with the University of Veterinary Medicine, Budapest. The size of the right prescapular lymph node (at least in one dimension) was measured using a caliper to estimate the remission rate. Complete remission (CR) was defined by an unpalpable/normal size prescapular lymph node (remission of lymph node size by 100%).

#### 2.1.1. Biochemistry and Hematology Examinations

Routine hematological parameters were examined weekly using a Sysmex XT-2000 iV™ (Sysmex, Kobe, Japan) hematology analyzer with veterinary software and the plasma biochemical parameters were measured every third week by a clinical chemistry analyzer (Olympus 460, Beckman Coluter) [40]. Qualitative blood count was performed by the microscopy analysis of blood smears.

#### 2.1.2. Evaluating Adverse Drug Effects

Recommendations of the Veterinary Cooperative Oncology Group (VCOG-CTCAE) were used to grade adverse drug effects [41]. 

#### 2.1.3. Lymph Node and Bone Marrow Sample Collection and Preparation

Samples from the lymph node and bone marrow were collected under general anesthesia, which was carried out with propofol 5 mg/kgbw iv, isoflurane 1.5–2.5 V/V%, and fentanyl by constant rate infusion of 0.01 to 0.04 mg/kgbw/h For routine histological and immunohistochemical examination, an enlarged lymph node was excised, whereas for cytological analysis, bone marrow aspirates were taken with a Jamshidi needle from the iliac crest (crista iliaca externa). For cytological evaluation, the aspirates were smeared and stained with a panoptic staining kit (REAGENS Ltd., Budapest, Hungary). 

Sections of (4%, neutralized) formalin-fixed paraffin-embedded lymph node tissues were kept at 56 °C for 12 h in an incubator to prevent the detachment of sections from the silanized slides during antigen retrieval in the immunohistochemistry procedure. The conventional panoptic (May-Grünwald and Giemsa, Sigma-Aldrich, St. Louis, MO, USA) procedure was used to stain the bone marrow.

### 2.2. Histopathology and Immunohistochemistry

The 3 μm thick serial sections were stained with hematoxylin and eosin (HE) for histological evaluation. CD3 was used as a T-cell marker (rabbit anti-CD3 DAKO, High Wycombe, UK) and CD79a for B-cell labelling (mouse anti-CD79a DAKO, High Wycombe, UK), as described by Kiupel et al. [42]. Tumors were graded based on the REAL classification scheme [43] applied for canine lymphoma [8,44], while staging was performed according to Valli et al. [8]. Proliferation status of the tumor was assessed by immunostaining against Ki67 (Monoclonal mouse MIB-1 anti-Ki-67: DAKO Ltd., High Wycombe, UK). To assess the percentage of the Ki67 positive cells, approximately 100 cells were counted in five different fields on each slide [45].

### 2.3. Calcein AM Assay

Measurements were performed at the time of diagnosis. Lymph node samples were cut into small pieces and immersed into dissociation medium containing Dulbecco’s modified Eagle medium (DMEM), 200 U/mL collagenase type II, and 0.6 U/mL dispase (Gibco, Life Technologies, New York, NY, USA). Following a 30-min-long incubation at 37 °C, a single cell solution was prepared by filtering the dissociated tumor through a 40 µm cell strainer. Isolated cells were centrifuged at 300× *g*. Samples (750.000 cells in 300 µL DMEM) were preincubated for 5 min at 37 °C in the presence of 10 μM verapamil (Sigma) [46] or 0.5 µL DMSO. The reaction was started by the addition of 0.25 µM calcein acetoxymethyl ester (Calcein AM) (Invitrogen, Life Technologies, USA). Cells were incubated for 10 min at 37 °C. The reaction was stopped by 1 mL cold (4 °C) PBS and 5 min centrifugation at 300× *g*; the cell pellets were resuspended in 270 µL PBS containing 0.8 μL 7-AAD (7-aminoactinomycin D 1 mg/mL, Sigma-Aldrich, USA). Samples were stored on ice and measured within 4 h with a FACScan flow cytometer (Becton Dickinson Biosciences, San Jose, CA, USA). Based on the quantity of the tissue samples, two to three parallel measurements were performed from each sample and the mean value was reported.

### 2.4. FACS Analysis

Identification of the lymphoid cells was based on the size and granularity. Dead cells were excluded based on 7-AAD staining. A total of 10,000 viable cells were analyzed. To represent P-gp activity, the dimensionless multidrug resistance activity factor (MAF) value was used, which was shown to correlate with the P-glycoprotein mediated drug efflux function [47]. MAF values were determined using the mean fluorescence intensity measured in the presence and absence of verapamil (mean fluorescence inhibited, MFI; and noninhibited, MFNI respectively): MAF = (MFI − MFNI)/MFI, as previously described [48].

### 2.5. Statistical Analysis

We compared the groups by one-way randomized block analysis of variance. In the case of significant *p* values (<0.05), we performed Bonferroni comparisons with different multiple pairings of the groups (1–2, 2–3, 1–3, etc.). The Student *t*-test (double sided, non-paired, with non-equal variance) in Excel (Microsoft^®^ Excel^®^ for Office 365 (16.0.12527. 22,180 for 64 bytes) was also used to compare the samples in multiple pairs (1–2, 2–3, 1–3, etc.). For survival analyses, we used the Log-rank, Cox-proportional hazard regression, and Kaplan–Meyer analyses, and similarly to the other comparisons, we performed the analyses by pairing the groups (1–2, 2–3, 1–3, etc.). Multiple Khi square tests were used to evaluate the frequency of a parameter in different groups. The relapse free period (RFP) and overall survival times (OST) of the different groups were compared, and the differences were considered significant at *p* < 0.05. 

Microsoft Excel 2010, R version 3.0.20 (Foundation for Statistical Computing, Vienna, Austria) and Stats Direct Statistical Software version 3.0.194 (StatsDirect Ltd., Altrincham, UK) were used for statistical analysis and the Reference guide for Reference Value Advisor v2.1. (National Veterinary School of Toulouse) was used for the normal range calculation. The “extent of exposure to risk of death” refers to the number of expected deaths. “Relative rate” compares the risk of a health event (disease, injury, risk factor, or death) between one group with the risk (exposed) with another group (unexposed).

## 3. Results

### 3.1. Clinical Characteristics

A total of 79 canine B-cell lymphoma patients were included in this study (44 males and 35 females). The mean age of the dogs was 8.2 years (1 to 5 years n = 15, 5–10 years n = 48, 10–18 years n = 16). 

Cytology and flow cytometric immunophenotyping were performed on all patients. Histological examination and immunohistochemistry were performed in 68 cases. Lymph node samples were collected during surgical biopsy under general anesthesia. Histopathological characteristics of the 68 patients were as follows: diffuse large B-cell type lymphoma (n = 56), among these two were T-cell rich B-cell lymphoma (n = 2). The remaining dogs were diagnosed with B-cell small lymphocytic lymphoma (n = 3), B-cell immunoblastic lymphoma (n = 7), and marginal zone lymphoma (n = 2). In terms of stages, there was only one case in Stage II, other stages included Stage III (n = 12), Stage IV (n = 52), and Stage V (n = 14). A total of 58 and 21 dogs were classified into Substage “a” and “b”, respectively. In addition to immunohistochemistry, the proliferation marker Ki 67% was determined in the surgically excised lymph nodes. The mean of the positive cells in the examined histological sections was 41.40%.

A total of 76 patients were treated with the CHOP protocol (Table 1), and three dogs received L-CHOP in the first line treatment (Table 2). In the CHOP protocol, first relapse occurred within 19 weeks in 19 cases. Chemotherapy was continued after the first relapse in 26 cases. The CHOP protocol was continued (n = 10) or replaced by L-CHOP (n = 10), or L-asparaginase and lomustine combined rescue treatment (n = 6).

Lymphoma related death was noted in 52 dogs and death due to other reasons (i.e., accidents, infection, trauma etc.) in 27 cases. Chemotherapy was postponed due to adverse effects in 33 cases. More prominent adverse reactions, namely anorexia (n = 48), neutropenia (n = 48), sterile hemorrhagic cystitis (n = 6), emesis (n = 47), and diarrhea (n = 30) were noted altogether in 63 occasions. Inappetence and other less remarkable and short duration side effects such as thrombocytopenia, fever, etc. were not detected. Grades of different prominent adverse reactions were registered according to VCOG-CTCAE (2021) grades “0” (n = 16), “1” (n = 26), “2” (n = 18), “3” (n = 11), “4” (n = 6), and “5” (n = 2); the statistical analyses considered only the highest grade events. Most dogs with adverse reactions (n = 63) had at least one of four side effects (neutropenia, cystitis, emesis, diarrhea). A total of 44 dogs experienced multiple side effects during the same 19 weeks of chemotherapy cycle (two: n = 22, three: n = 21, and four side effects: n = 1). Prednisolone and antibiotics were administered before complete diagnosis by the referring veterinarian in 18 and 25 cases, respectively. Alterations in drug administration and dosing were noted during the first 19-week treatment cycle in all patients (1501 drug administrations). Drug administration was delayed due to side effects in 33/79 cases. These patients received the delayed treatment a week later. Altogether, 48/79 dogs did not receive treatment in time (125/1501 administrations), and 15/79 dogs did not obtain further therapy for other reasons. The mean grade of side effects in all dogs was 1.63. Between a 10 and 20% dose reduction was necessary in 16/79 dogs and 1/79 dog was treated once by 60% of the dose. Overall dose reduction was realized in 48/1501 treatments. Relapse occurred in 19/79 cases during the first cycle (19 weeks). The median overall survival time (OST) was 417 and the median relapse free period (RFP) was 301 days. Considering lymphoma-related death, the median OST was 348 days, and lymphoma-related deaths resulted in a median OST of 424 days. 

### 3.2. Correlations between Clinical Stages and Survival Times

**Figure 1:**
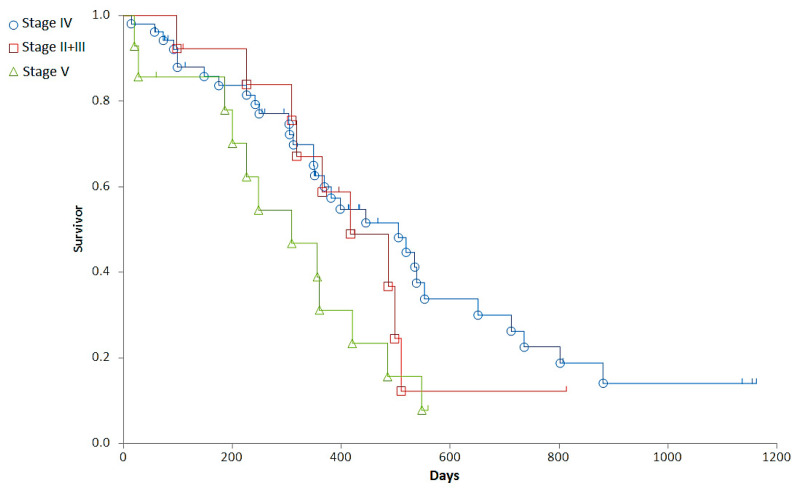
Representation of the overall survival times (OST) of dogs with different stages. Stage II + III (red squares), Stage IV (blue circle), Stage V (green triangle).

**Figure 2:**
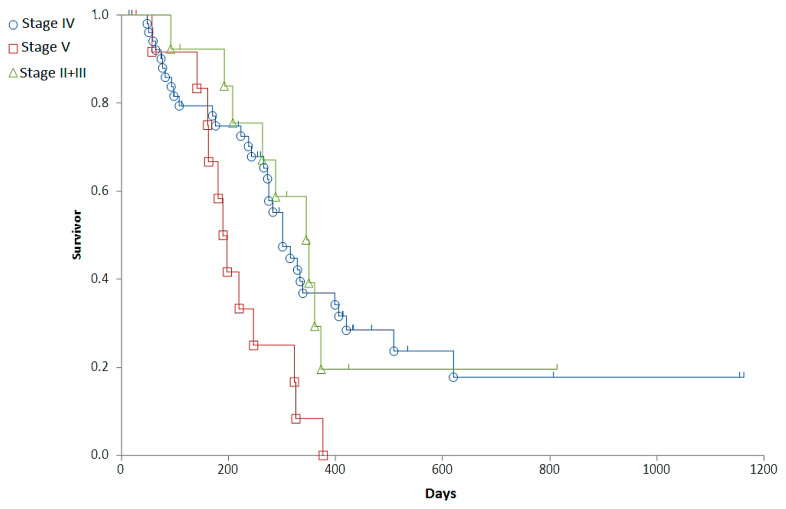
Representation of the relapse free period (RFP) of dogs with different stages on survival curves. Stage II + III (red squares), Stage IV (blue circle), Stage V (green triangle).

Staging was associated with a major difference in OST (Generalized Wilcoxon, Peto-Prentice: Chi-square for equivalence of death rates = 5.276997, *p* = 0.0715; Chi-square for trend 1.278467, *p* = 0.2582) (Table 3, Figure 1) and a significant difference in RFP (Generalized Wilcoxon, Peto-Prentice: Chi-square for equivalence of death rates = 8.874787, *p* = 0.0118; Chi-square for trend = 0.222321, *p* = 0.6373) (Table 4, Figure 2).

In summary, with Stage IV dogs having a longer RFP and OST than dogs in other stages, survival between Substage “a” (n = 58) and “b” (n = 21) was not significantly different, despite a clear tendency of shorter survival times in Substage “b” (median OST was 485 and 349 days, and RFP was 301 and 237 days in Substage “a” and “b”, respectively). The OST and RFP showed significant (*p* < 0.05) inverse correlation with the VCOG-CTCAE grade of the most remarkable side effects (r = −0.3019 and −0.2232, respectively).

### 3.3. Evaluation of P-gp Activity

#### 3.3.1. Determination of the Cut off MAF Value

**Figure 3:**
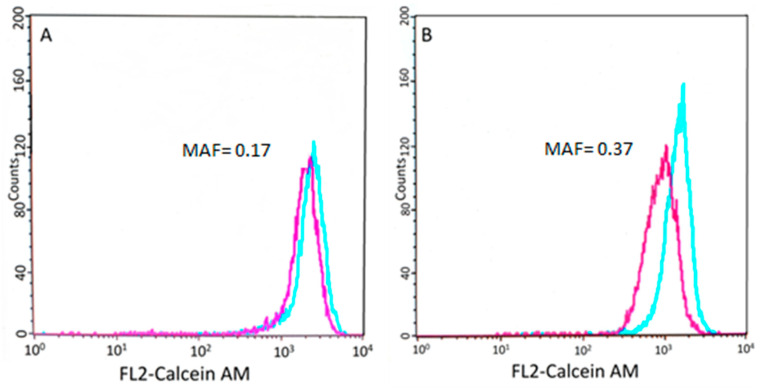
Results of the Calcein AM assay. Flow cytometry histogram showing the calcein fluorescence of tumor cells incubated in the presence (blue) or absence (purple) of verapamil, an inhibitor of the efflux pump (see Section 2). (**A**) Cells isolated from patient 5148–5570 show low P-gp activity. (**B**) Conversely, cells isolated from patient 9865–10,531 show lower fluorescence, which was restored by the P-gp inhibitor verapamil.

P-gp-mediated drug resistance was characterized using the Calcein assay, performed on tumor cells isolated from lymph node biopsies at the time of diagnosis. Drug resistance was quantified by the multidrug resistance activity factor (MAF), a dimensionless value ranging from 0 to 1 (Figure 3).

**Figure 4:**
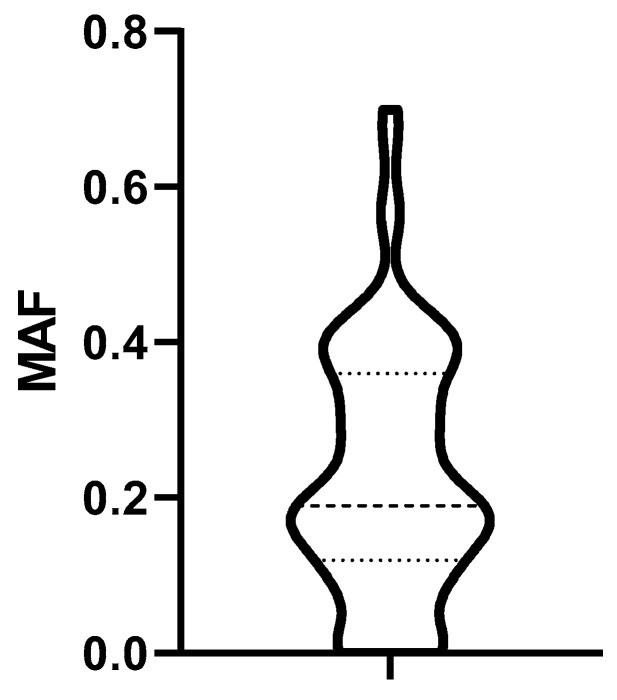
Distribution of MAF values. Dashed lines represent the median, the 25% and the 75% percentile. MAF: multidrug resistance activity factor.

The distribution of MAF values characterizing P-gp activity of biopsied lymphoma cells obtained from 79 dogs at diagnosis is shown in Figure 4.

MAF in Stage V patients were the highest, but a comparison of the MAF values in different stages did not reveal significant differences (Table 5). The treatment-naive lymph nodes of dogs in Substage “a” had non-significantly higher MAF values than dogs belonging to Substage “b” (Table 6).

**Figure 5:**
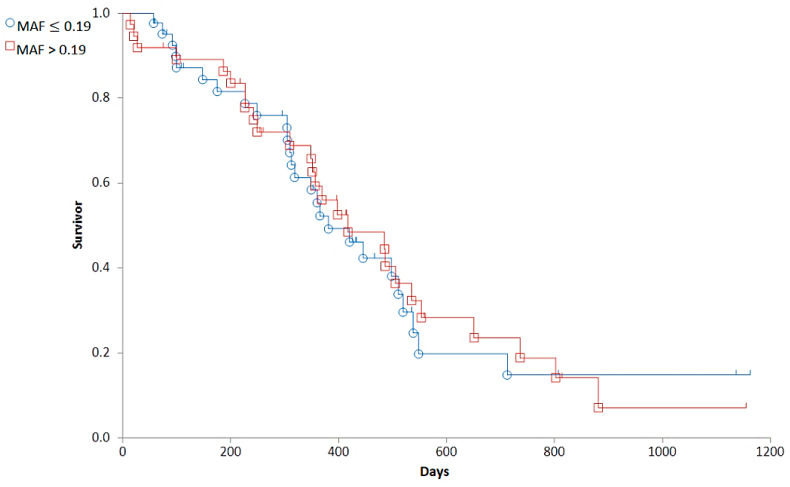
The overall survival of canine lymphoma patients with MAF ≤ 0.19 (blue, circle) and MAF > 0.19 (red, squares).

**Figure 6:**
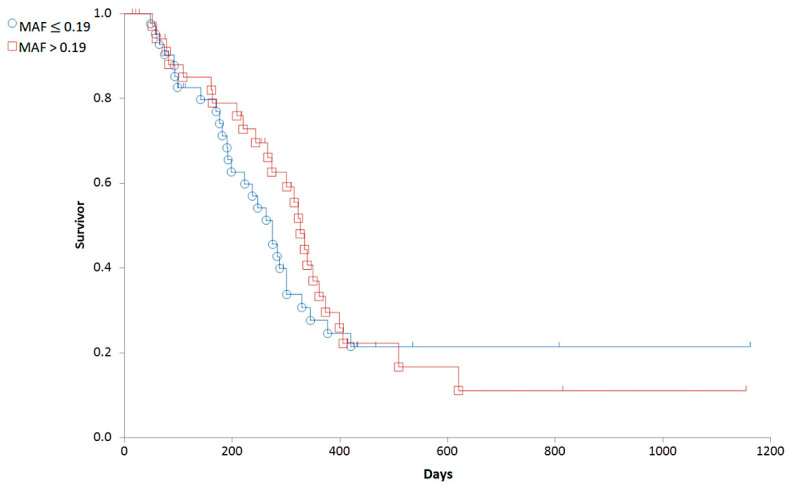
Relapse free survival of canine lymphoma patients with MAF ≤ 0.19 (blue circle) and MAF > 0.19 (red squares).

Based on the distribution of MAF values (Figure 4), we established a cut-off corresponding to the median (0.19). To determine the predictive value of the Calcein test for the outcome of the therapy, the 79 patients were divided into two groups corresponding to low (MAF ≤ 0.19; n = 42) and high (MAF > 0.19; n = 37) P-gp activity. In contrast to our expectations, there was no significant difference in the OST of the two groups (median OST = 381 days for dogs with MAF ≤ 0.19; median OST = 417 days for dogs with MAF > 0.19; *p* = 0.8897; Figure 5). Interestingly, patients with MAF ≤ 0.19 showed a tendency of an earlier relapse (median RFP = 275) compared to dogs with MAF > 0.19 (median RFP = 326 days; Log rank: *p* = 0.5878, respectively, Figure 6). P-gp activity showed no correlation with survival regardless of the cut-off value or whether MAF was considered as a continuous variable. Taken together, these results indicate that P-gp activity, as determined by the Calcein assay performed on tumor cells obtained at diagnosis, does not predict therapy response and survival in canine B-cell lymphoma. 

The lack of correlation between P-gp activity and survival may be due to confounding factors. To analyze the relation of MAF and other clinical parameters recorded in the study, dogs were grouped into four categories, based on the median RFP (301 days) and MAF (0.19) values. The expected relation between P-gp activity and survival (RFP and OST) could be established in the case of only 34 patients (43.03%) (Group 1). We further divided this group into subgroups with high RFP (>301 days) and low MAF (≤0.19) (+/−, n = 13) (Group 1a), and with low RFP (<301 days) and high MAF (>0.19)(−/+, n = 21) (Group 1b). Dogs in Group 1a with MAF ≤ 0.19 lived longer (median OST = 538 days) and showed later relapse (median RFP = 484) than the patients with MAF >0.19 in Group 1b (median OST = 279 days; median RFP = 220 days (Log rank: *p* < 0.0004, *p* < 0.0001, respectively). On the other hand, survival of the remaining 45 dogs in Groups 2 and 3 was not related to MAF values as there were dogs with low initial MAF values that nevertheless produced short survival times (low RFP, OST and low MAF (−/−, n = 29) (Group 2)) as well as dogs with high MDR activity at diagnosis showing long survival (high RFP, OST, and high MAF (+/+, n = 16) (Group 3)). Group 2 showed shorter RFP (less than 301 days) despite having MAF ≤0.19 (n = 29), while in Group 3, there were dogs with MAF > 0.19 and still reaching a RFP longer than 301 days (n = 16); OST followed the same trends. 

#### 3.3.2. Correlations between MAF and Clinical Variables

To account for the influence of clinical variables, groups 1–3 defined above were compared with respect to the adverse reactions, modification of the therapy protocols and the cause of death. As a measure of the adverse reactions, unfavorable and unintended symptoms were graded according to the Veterinary Cooperative Oncology Group common terminology criteria (VCOG-CTC). As expected, survival times were negatively correlated with the severity of adverse effects. Interestingly, MAF values also showed a significant (*p* < 0.05) inverse correlation with the VCOG-CTCAE grade of side effects, and with the frequency of slipped drug administrations (Table 7). 

Therapy was delayed due to side effects significantly more often in Group 1 (58.6%) than in Group 2 (25%). The grade of different side effects (VCOG-CTCAE) was evaluated by recording the most remarkable side effects during treatment (vomiting, diarrhea, cystitis and neutropenia). Dogs in Group 1 showed significantly more severe side effects (mean grade: 2.21) than dogs in Group 2 (mean grade: 1.13) or Group 3 (mean grade: 1.38) In some cases, the manifestation of side effects led to the reduction in the dose, however, the groups were similarly affected (Table 8). Dogs in Group 1 showed relapse more often within the first cycle (48.3%) than dogs in Group 2 (6.25%) or Group 3 (11.7%) (Table 9). 

Another confounding factor may be the cause of death. The rate of lymphoma-related death did not show significant differences between the groups (Group 1: 0.69 (n = 20/29); Group 2: 0.63 (n = 10/16); Group 3: 0.65 (n = 22/34)). Similarly, there was no difference in Ki67-positivity. The mean Ki67 positivity was 41.4% (SD ± 13.57). In summary, these results indicate that the lack of the correlation between the initial P-gp activity measured in treatment-naïve tumor cells and therapy outcome cannot be explained by confounding clinical parameters. 

## 4. Discussion

In the current study, biopsy samples obtained from canine patients were used to investigate the predictive value of P-gp function at the time of diagnosis. The characteristics of our patient cohort in terms of stages [41], therapeutic response to chemotherapy, and histologically determinable subtypes within the B phenotype canine lymphomas [43] were according to the reported standards. Treatment efficacy regarding the survival time of our patients who died due to lymphoma (median: 348 days) is comparable with reported results [4]. The estimated median RFP and lymphoma related survival time for all dogs were 414 days (95% confidence interval (CI), range 228–600 days) and 442 days (95% CI, range 236–648 days), respectively, in a study published by Pioggi et al. [49]. Another study reported 121 cases treated by chemotherapy, with a median survival time (MST) of 300 days (range 1–1644 days) [50]. In yet another study, median RFP was 196 days (range 22–1656 days) and OST was 292 days (range 40–2246 days) of dogs receiving CHOP (cyclophosphamide, doxorubicin, vincristine, and prednisolone) based protocols [51]. The time of first relapse (RFP) is an accurate indicator of survival statistics because it is not affected by variables influencing OST (such as non-lymphoma related diseases, for instance, accidents, heart disease, infections). In our study, we observed similar RFPs as reported earlier by others [8,9]. The most important factor influencing RFP is the occurrence of side effects, which cause delays in the chemotherapy protocol [52].

Several studies have been published on the evaluation of P-gp expression by immunohistochemistry [10,23,25], Western blotting [26], flow cytometry [28,53], and reverse transcription polymerase chain reaction (RT-PCR) [54]. Flow cytometry enables the evaluation of transporter function in single cells based on the efflux of fluorescent dyes [28]. Rhodamine 123 has been repeatedly used for the evaluation of P-gp function in canine lymphocytes and lymphoma cells [11,28,54]. Although the sensitivity of this method is high, proper quantification of the transport activity is hindered by the relatively poor cellular retention of rhodamine and its interaction with various intracellular compartments and organelles such as mitochondria [55,56]. Based on our earlier work conducted with human acute myeloid leukemia patients [30], in this study, we used the Calcein assay to measure the P-gp activity in tumor cells. 

Based on in vitro studies, MAF values showed excellent correlation with the P-gp levels in cell lines [56]. Clinical studies have suggested that MAF values above 20% show a high predictive value for the therapy failure of AML patients [30]. In our study, treatment-naïve lymphoma cells showed a wide distribution of MAF values ranging from 0 to 0.551. There were no significant differences between the MAF values of patients with different stages. Based on the known role of P-gp mediated drug efflux in multidrug resistance, we expected that higher MAF values would be correlated with shorter survival times. However, our data do not support this expectation, as in many dogs, low MAF values were associated with short survival times, and some dogs with high initial MAF values produced long survival times. Insufficient sample size and the combined analysis of heterogeneous B-cell lymphoma subtypes are not likely to be the cause of this negative result, as our study was based on a sufficiently large patient cohort with well-defined subtypes, covering a relatively long time period (7 years). 

A potential confounding factor may be that several dogs were euthanized without a veterinary indication (based on the owner’s request), and these patients had to be censored in the survival analysis, which obviously impacted the statistical outcome of the study. From a technical perspective, the Calcein assay can be considered as a highly reproducible and robust assay to quantify P-gp function. Tumor sampling was performed from the lymph node, and in some cases, samples were taken by needle biopsy in a transcutaneous way. Accordingly, the assay results may have been influenced by the ratio of neoplastic and microenvironmental cells [57,58].

Adverse reactions may require chemotherapy dose adjustment that can mask the influence of factors contributing to resistance [35]. Interestingly, during the first 19 weeks of therapy, dogs in Group 2 (MAF ≤ 0.19) tended to be more susceptible to adverse drug reactions and the frequency of postponed drug administrations was also inversely correlated with the MAF values (Table 8). The decreased survival times of dogs in Group 2 can be explained either by an increased sensitivity to drugs or with the emergence of other, possibly non transporter mediated drug resistance mechanisms. Dogs in Group 2 showed significantly more severe side effects due to chemotherapy, frequently causing postponed drug administration times. These observations, summarized in Table 8 and Table 9, may suggest that low P-gp activity in tumor cells indicate an increased sensitivity to cytotoxic drugs. However, low P-gp function was also present in Group 1a, where the longest OSTs were observed. More likely, lymphomas in Group 2 are characterized by different resistance mechanisms, which could explain the shorter RFP and OST. 

Thus, shorter survival times may be explained, at least in part, by an increased sensitivity to drugs and the ensuing changes of the treatment schedules. We reported similar findings previously using immunohistochemistry for P-gp detection [34]. In that study, lower P-gp expression levels were associated with a higher probability of death due to adverse drug reactions. That the efflux function of tumor cells is indicative of the general sensitivity of microenvironmental cells was suggested by another study, which showed that neutropenia of canine lymphoma patients during chemotherapy was associated with the diminished resistance of tumor cells, and less severe side effects were associated with higher levels of drug resistance. However, these correlations did not influence the overall survival as neutropenic patients lived longer than those without neutropenia [59]. Yet another study has shown that basal and follow-up ABCB1 gene expression levels predicted the occurrence of severe adverse drug reactions [60]. In general, therapy-related toxicity depends on the organism’s ability to systematically detoxify cytostatic drugs. As adverse effects and P-gp activity in tumor cells are unlikely to be causally linked, these results may suggest that P-gp activity in cancer cells may be a surrogate marker of the patient’s capacity to excrete P-gp substrates through hepatic, renal, or intestinal clearance. However, this hypothesis would need further testing.

We cannot exclude the confounding effects related to the treatments. An inverse relation between P-gp activity and survival may be expected if the chemotherapeutic compound is recognized by P-gp. Lymphoma protocols contain drugs, which are susceptible to P-gp mediated efflux such as vinca alkaloids (vincristine, vinblastine) and doxorubicin [61,62]. Differences in prior treatment histories may have also modified the MDR phenotype due to the administration of P-gp substrates such as tetracycline, ivermectin, or corticosteroids (methylprednisolone, dexamethasone, cortisol) [63]. Moreover, several compounds act as P-gp-inhibitors including azithromycin, clarithromycin, erythromycin, omeprazole, pantoprazole, esomeprazole—all of which are often used drugs in canine lymphoma treatment [64]. 

Tumors derived from tissues with high levels of membrane efflux transporter expression are often drug resistant (e.g., colon, kidney, pancreas, and liver carcinoma). In humans, high P-gp expression was observed in several hemopoietic malignancies (leukemia, lymphoma, multiple myeloma) and some carcinomas [13]. In some tumor types including hemangiopericytoma, apocrine gland adenocarcinoma, hepatoma, cholangiocarcinoma, transitional cell carcinoma, adrenal gland adenoma, colorectal adenoma, and adenocarcinoma, more than 50% of the canine cases were positive for P-gp expression as determined by immunohistochemistry. A study found that 27.3% of lymphoma cases expressed P-gp [10]. However, intrinsic P-gp expression can be modified, inhibited, or induced by various natural products such as 5′-methoxyhydnocarpin-D, which is a natural MDR inhibitor produced by Berberis plants [65]. In addition, the tumorous lymph node tissues characterized by low initial P-gp expression may display an increased efflux function later, as a result of an induction by chemotherapy. While the induction of P-gp could have a significant influence on therapy response, our study measuring intrinsic P-gp expression levels in treatment-naïve tumor cells was not designed to assess the relevance of acquired drug resistance. 

We have limited information regarding ABC transporters in canine cancer patients as most of our knowledge is based on in vitro studies. There are very few clinical trials assessing the relevance of the expression and function of these transporters in canine patients. Our study did not analyze the contribution of other ABC-transporters associated with drug resistance such as the multidrug resistance-associated protein 1 (MRP1) and 2 (MRP2) and breast cancer resistance protein (BCRP) [66,67]. Other mechanisms known to cause therapy resistance such as the cytochrome P450 system [68] or antioxidants [69] may have also blunted the effect of intrinsic P-gp activity on the outcome of the therapy.

## 5. Conclusions

Taken together, our study shows that Pgp activity measured at diagnosis does not predict therapy outcome in canine B-cell lymphoma, regardless of the immunophenotype of the cancer cells. In our study, we used the Calcein assay to quantify the intrinsic P-gp activity in tumor cells before the administration of cytotoxic chemotherapy. It will be interesting to characterize the changes of P-gp function along the course of chemotherapy to identify whether drug efflux mediated by P-gp contributes to acquired resistance, thus limiting the treatment of canine lymphoma patients.

## Figures and Tables

**Table 1:** The CHOP treatment protocol.

	Chemotherapy	Additional Therapy
Week 1	Doxorubicin (30 mg/m^2^ iv.)	Prednisone (2 mg/kgbw/day po.)
Week 2	Vincristine (0.75 mg/m^2^ iv.)	Predinsone (1.5 mg/kgbw/day po.)
Week 3	Vincristine (0.75 mg/m^2^ iv.)	Prednisone (1 mg/kgbw/day po.)
Week 4	Vincristine (0.75 mg/m^2^ iv.) Cyclophosphamide (250 mg/m^2^ po.)	Prednisone (0.5 mg/kgbw/day po.)
Week 5	Vincristine (0.75 mg/m^2^ iv.)	
Week 6	Vincristine (0.75 mg/m^2^ iv.)	
Week 7	Vincristine (0.75 mg/m^2^ iv.) Cyclophosphamide (250 mg/m^2^ po.)	
Week 8	Vincristine (0.75 mg/m^2^ iv.)	
Week 9	Vincristine (0.75 mg/m^2^ iv.)	
Week 10	Doxorubicin (30 mg/m^2^ iv.)	
Week 11	Vincristine (0.75 mg/m^2^ iv.)	
Week 12	Vincristine (0.75 mg/m^2^ iv.)	
Week 13	Vincristine (0.75 mg/m^2^ iv.) Cyclophosphamide (250 mg/m^2^ po.)	
Week 14	Vincristine (0.75 mg/m^2^ iv.)	
Week 15	Vincristine (0.75 mg/m^2^ iv.)	
Week 16	Vincristine (0.75 mg/m^2^ iv.) Cyclophosphamide (250 mg/m^2^ po.)	
Week 17	Vincristine (0.75 mg/m^2^ iv.)	
Week 18	Vincristine (0.75 mg/m^2^ iv.)	
Week 19	Doxorubicin (30 mg/m^2^ iv.)	

**Table 2:** The L-CHOP protocol.

	Chemotherapy	Additional Therapy
Week 1	L-Asparaginase (400 IU/kgbw im.) Vincristine (0.75 mg/m^2^ iv.)	Prednisone (2 mg/kgbw/day po.)
Week 2	Cyclophosphamide (250 mg/m^2^ po.)	Predinsone (1.5 mg/kgbw/day po.)
Week 3	Vincristine (0.75 mg/m^2^ iv.)	Prednisone (1 mg/kgbw/day po.)
Week 4	Doxorubicin (30 mg/m^2^ iv.)	Prednisone (0.5 mg/kgbw/day po.)
Week 5	No medication	
Week 6	Vincristine (0.75 mg/m^2^ iv.)	
Week 7	Cyclophosphamide (250 mg/m^2^ po.)	
Week 8	Vincristine (0.75 mg/m^2^ iv.)	
Week 9	Doxorubicin (30 mg/m^2^ iv.)	
Week 10	No medication	
Week 11	Vincristine (0.75 mg/m^2^ iv.)	
Week 12	No medication	
Week 13	Cyclophosphamide (250 mg/m^2^ po.)	
Week 14	No medication	
Week 15	Vincristine (0.75 mg/m^2^ iv.)	
Week 16	No medication	
Week 17	Doxorubicin (30 mg/m^2^ iv.)	
Week 18	No medication	
Week 19	Vincristine (0.75 mg/m^2^ iv.)	
Week 20	No medication	
Week 21	Cyclophosphamide (250 mg/m^2^ po.)	
Week 22	No medication	
Week 23	Vincristine (0.75 mg/m^2^ iv.)	
Week 24	No medication	
Week 25	Doxorubicin (30 mg/m^2^ iv.)	

**Table 3:** Clinical stages of the patients at the time of diagnosis and their median overall survival times. The highest median OST was observed in dogs with Stage IV, which was also the group with the highest number of patients.

Stage	II + III	IV	V	*p*-Values
Number	13	52	14	
Observed deaths	9	31	12	
Extent of exposure to risk of death	8.602	36.705	6.693	
Relative rate	1.046	0.845	1.793	
Median OST	417	505	309	
II + III vs. IV				0.5159
II + III vs. V				0.2356
IV vs. V				0.0233

**Table 4:** Clinical stages of the patients at the time of diagnosis and their median relapse free periods. The highest median RFP was observed in dogs with Stage II + III.

Stage	II + III	IV	V	*p*-Values
Number	13	52	14	
Observed deaths	9	32	12	
Extent of exposure to risk of death	10.863	36.629	5.506	
Relative rate	0.828	0.874	2.179	
Median RFP	345	301	190	
II + III vs. IV				0.8815
II + III vs. V				0.0206
IV vs. V				0.0062

**Table 5:** A comparison of the MAF values in different stages.

	Stage II + III	Stage IV	Stage V
Number	13	52	14
Mean MAF	0.205	0.236	0.233
SD	±0.109	±0.175	±0.133
*p*-value	One way ANOVA global test: *p* = 0.8231		

**Table 6:** A comparison of the MAF values in different substages.

MAF	Substage “a”	Substage “b”
Number	58	21
Mean	0.235	0.216
Standard deviation	0.164	0.149
*p*-value	One way ANOVA, global test: *p* = 0.6559	

**Table 7:** A comparison of the grade of side effects with MAF and survival times.

Variable (1)	Variable (2)	Pearson R-Value	Linear Regression, Kendall’s Rank Correlation Coefficient	*p*-Value
MAF	Grade of side effects	−0.3347	−0.258	0.0025
MAF	Slipped drug administration time due to side effect	−0.2734	−0.264	0.0052
OST	Grade of side effects	−0.3019	−0.235	0.0052
RFP	Grade of side effects	−0.2232	0.214	0.0111

**Table 8:** A comparison of patient groups with respect to alteration of the treatment regimen due to adverse drug reactions, anorexia, and grade of side effects.

Observed Parameters	Group 1	Group 2	Group 3	*p*-Value
**Slipped drug administration time**				
Occasion/Number	12/34	17/29	4/16	
%	35.3	58.6	25.0	
Group 1 vs. Group 2				0.0641
Group 1 vs. Group 3				0.4667
Group 2 vs. Group 3				0.0304
**Dogs with dose reduction**				
Occasion/Number	6/34	8/29	3/16	
%	17.64	27.58	18.75	
Group 1 vs. Group 2				0.344254
Group 1 vs. Group 3				0.924557
Group 2 vs. Group 3				0.509106
**Grade of side effects**	**Group 1**	**Group 2**	**Group 3**	***p*-value**
Number	34	29	16	
Mean	1.38	2.21	1.13	
± SD	1.26	1.35	0.78	
Group 1 vs. Group 2				0.0173
Group 1 vs. Group 3				0.3925
Group 2 vs. Group 3				0.0018
**Dogs with anorexia**				
Occasion/Number	18/34	21/29	9/16	
%	52.94	72.41	56.25	
Group 1 vs. Group 2				0.1126
Group 1 vs. Group 3				0.8266
Group 2 vs. Group 3				0.2708

**Table 9:** A comparison of the three created groups in terms of early (during first cycle of treatment) relapse.

Relapse during the First 19 Weeks	Group 1	Group 2	Group 3	*p*-Value
Occasion/Number	4/34	14/29	1/16	
%	11.7	48.3	6.25	
Group 1 vs. Group 2				0.0014
Group 1 vs. Group 3				0.5443
Group 2 vs. Group 3				0.0042

## Data Availability

The data presented in this study are available in this article.
